# Differential Fixation and Eye Alignment Patterns in Strabismus with and Without Amblyopia Across Viewing Conditions

**DOI:** 10.3390/jemr19030047

**Published:** 2026-05-03

**Authors:** Archayeeta Rakshit, Ibrahim M. Quagraine, Gokce Busra Cakir, Aasef G. Shaikh, Fatema F. Ghasia

**Affiliations:** 1 Ocular Motility & Vision Neurosciences Laboratory, Cole Eye Institute, Cleveland Clinic, Cleveland, OH 44195, USA; rakshia@ccf.org (A.R.); gokcebusracakirmd@gmail.com (G.B.C.); 2 Department of Biomedical Engineering, Case Western Reserve University, Cleveland, OH 44195, USA; imq3@case.edu; 3 Daroff-Dell’Osso Ocular Motility Laboratory, Louis Strokes Cleveland VA Medical Center, Cleveland, OH 44195, USA; axs848@case.edu

**Keywords:** amblyopia, strabismus, fixation instability, fast and slow FEM, eye alignment

## Abstract

Fixation instability (FI) and vergence instability (VI) in amblyopia and strabismus are associated with disrupted physiologic fixation eye movements (FEMs). This study examined how viewing conditions affect FEM patterns in strabismic subjects with and without amblyopia. FEMs of the non-dominant/amblyopic and dominant/fellow eyes were recorded using video-oculography during both-eye viewing (BEV), fellow/dominant-eye viewing (FEV/DEV), and amblyopic/non-dominant-eye viewing (AEV/NDEV) in strabismic subjects with amblyopia (SA, n = 56), without amblyopia (S, n = 19), and controls (C, n = 25). FI, VI, fast FEM amplitudes, slow FEM velocities, and time-based control of eye deviation were analyzed. The SA group showed the greatest FI in the amblyopic eye during AEV compared with the fellow eye during FEV, whereas minimal inter-ocular FI differences were observed in the S group and controls. Under monocular viewing, both SA and S groups exhibited increased FI in the non-viewing eye and higher VI than controls. Regression analyses indicated that visual acuity deficits primarily influenced viewing-eye FI and FEM dynamics, while strabismus mainly affected non-viewing-eye FI and slow FEMs. C and S groups showed the least eye deviation during BEV, whereas the SA group showed the least eye deviation—but the highest VI—during AEV, indicating a distinct pattern of incomitance. Distinct FEM patterns shaped by viewing conditions may reflect underlying visuomotor control mechanisms and serve as biomarkers for AI (artificial intelligence)-based classification.

## 1. Introduction

Amblyopia is a neurodevelopmental visual disorder that arises from disruption of correlated activity between the two eyes—commonly due to anisometropia, strabismus, or visual deprivation [1]—during the critical period of visual development, resulting in reduced visual acuity in one eye [1,2,3,4]. Strabismus, defined by ocular misalignment, frequently can also occur without amblyopia, and individuals with strabismus with and without amblyopia often have reduced depth perception [stereoacuity] and interocular suppression [5]. These sensory deficits are often accompanied by oculomotor abnormalities, with fixation instability [FI] being one of the most consistently reported features [6,7,8,9,10,11,12,13,14]. FI in amblyopia and strabismus has been attributed to alterations in physiologic fixation eye movements [FEMs], including enlarged microsaccades and increased intersaccadic drifts and nystagmus—such as fusion maldevelopment nystagmus [8,11,12,13,14,15,16,17,18,19,20,21,22,23]. Under normal conditions, fixational eye movements (FEMs) generate controlled image motion within the foveola, supporting high-acuity vision and stable fixation [20,24,25,26,27,28,29]. Further, in healthy binocular vision, visual and oculomotor control mechanisms are well coordinated such that fixation is stable not only on the retina but also in depth, with fixation disparity remaining within fusion-tolerant limits defined by Panum’s fusional area [15,16,17,18,21]. In contrast, in amblyopia and strabismus, impaired interocular coordination and increased fixation instability lead to larger and more persistent fixation disparities that exceed fusion limits, resulting in degraded vergence control, increased vergence instability, and disruption of binocular function [10,13,14,20,22,23].

Previous studies have used the term “fixation instability” to describe oculomotor behavior of the non-viewing or occluded eye during attempted fixation; we follow this convention to maintain consistency with the established literature [30,31,32].

Our previous work has shown that fixation instability is associated with more severe visual function deficits in amblyopia, including reduced contrast sensitivity, decreased visual acuity in the amblyopic eye, impaired stereoacuity, and increased suppression [33,34]. Importantly, visual input and viewing conditions have been shown to modulate FEMs. For instance, FI in the fellow eye is more pronounced during monocular viewing with the amblyopic eye than during viewing with the fellow eye itself, indicating that viewing conditions differentially affect FI [9,14,35]. Additionally, studies using dichoptic paradigms—where the contrast of the fellow eye is reduced while the amblyopic eye views a high-contrast stimulus—have demonstrated that selective reduction of fellow-eye input increases its instability, further highlighting the role of visual input in modulating FEMs [34,36].

In this study, we investigate modulation of FEM abnormalities in individuals with strabismus, with and without amblyopia, across monocular and binocular viewing conditions. We quantified fixation and vergence stability, fast FEM amplitudes, slow FEM velocities, and the magnitude and control of eye deviation. Our goal was to identify oculomotor signatures that distinguish these clinical populations and to determine how viewing conditions modulate FEMs and ocular alignment. Visual acuity was measured using a psychophysical staircase procedure, and the eye with superior acuity was designated as the dominant eye, consistent with prior studies demonstrating associations between visual acuity, fixation instability, and fixation eye movements in amblyopia [6,7,8,9,14,37]. High-resolution video-oculography was used to analyze FEMs in the dominant/fellow eye and the non-dominant/amblyopic eye across three groups: controls, strabismic amblyopia, and strabismus without amblyopia under both eyes, dominant/fellow-eye, and non-dominant/amblyopic-eye viewing conditions. We hypothesize that visual input stabilizes fixation and vergence such that the absence of retinal error feedback leads to increased fixation instability in the occluded, deviated non-viewing eye in strabismus, whereas fixation instability persists in the viewing eye in amblyopia despite the availability of retinal error feedback due to reduced visual acuity. Furthermore, we predict that both-eye viewing will enhance vergence stability, while monocular viewing and the presence of strabismus will exacerbate VI. Finally, we anticipate that individuals with amblyopia will exhibit greater eye deviation during fellow-eye viewing compared to amblyopic-eye viewing, reflecting the combined effects of amblyopia and strabismus, whereas individuals with strabismus without amblyopia will not show such incomitance.

## 2. Materials and Methods

The study protocols were approved by the Cleveland Clinic Institutional Review Board, and written informed consent was obtained from each participant or their parent/legal guardian, in accordance with the Declaration of Helsinki. Verbal consent was also obtained from children less than 18 years. A total of 100 participants [40 males, 60 females) were recruited, comprising 25 healthy controls, 56 strabismus participants with amblyopia (SA), and 19 participants with strabismus without amblyopia (S). Subjects were classified as having strabismic amblyopia based on criteria established by the PEDIG studies [38,39,40]. All participants underwent comprehensive eye exams, including cycloplegic refraction, ocular motility assessment, strabismus evaluation, and stereoacuity measurements using the Titmus Stereoacuity Test. Stereopsis deficits were rated using the terms absent/gross stereopsis (i.e., absent and positive fly response pooled together), moderate stereopsis (800–100 arcsecs), fine stereopsis (better than 100 arcsecs). Control subjects were chosen based on the absence of any ocular or systemic abnormalities affecting visual acuity, except for refractive errors. Amblyopia was defined as reduced corrected distance visual acuity without structural abnormalities of the optic nerve, retina, or visual pathways. Subjects in the amblyopic cohort demonstrated (a) at least a 2-line difference in visual acuity between the two eyes at the time of diagnosis and had history of strabismus defined per previous PEDIG studies as heterotropia at distance (with or without spectacles), and/or (b) documented history of strabismus that is no longer present [39]. We recruited 19 strabismic subjects who had neither current amblyopia nor any history of treated amblyopia. The exclusion criteria included any coexisting ocular or systemic disease, congenital infections or malformations, and developmental delay. We also excluded subjects with idiopathic infantile nystagmus, characterized by an increasing-velocity slow-phase, or alternatively, pendular nystagmus [41,42].

### 2.1. Visual Acuity Measurement

Visual acuity was assessed using the ETDRS chart under best-corrected conditions following cycloplegic refraction, ensuring both eyes were optimally corrected and minimizing residual refractive error. Visual stimuli were generated using Psykinematix Version 2.4 (KyberVision Japan LLC) software on a 1280 × 800 resolution display at 60 Hz, with a white luminance of 111 cd/m^2^, viewed from 3.1 m in a dark room. Monocular visual acuity was assessed using ETDRS optotypes with crowding bars, with the non-viewing eye occluded. Testing was conducted in a randomized sequence for right and left eyes to avoid order effects. A 2-down-1-up staircase method adjusted optotype size based on responses, with step sizes decreasing by 50% before the first reversal, followed by 25% increments and 12.5% decrements across six reversals. Thresholds were calculated as the arithmetic mean of reversals and converted to logMAR [35]. In control and S group subjects, the eye with poorer acuity was designated as the non-dominant eye and the better-seeing eye as the dominant eye for analysis. As expected, there was no statistically significant difference in the measured visual acuity between the designated dominant and non-dominant eye in control and S subjects. However, this operational definition was chosen for analysis purposes, based on evidence linking fixation eye movements and acuity performance in amblyopia [6,7,8,14].

### 2.2. Fixation Eye Movement (FEM) Recordings

Binocular horizontal and vertical eye positions were recorded utilizing a high-resolution eye tracker (EyeLink 1000 plus; SR Research, Ottawa, ON, Canada) with 500 Hz sampling frequency and a spatial resolution of 0.01 [14]. Subjects wore corrective lenses if prescribed, while their heads were fixed on a chinrest positioned 84 cm away from a 30-inch LCD screen (2560 × 1600, 60 Hz, 350 cd/m^2^, Dell Technologies, Round Rock, Texas, United States). A forehead sticker was used to track head movements [43,44,45]. Monocular calibration and validation were performed for each eye individually using a 5-point horizontal and vertical target constellation [46]. During recordings, subjects fixated on a 0.5° white circular dot on a black background in a dark room. Three trials, lasting 45 s each, under both-eye viewing (BEV), fellow/dominant-eye viewing (FEV/DEV) and amblyopic/non-dominant-eye viewing (AEV/NDEV), were performed in a randomized order. For the monocular viewing, an infrared-permissive filter (Lee filters) was used to block visible light, enabling recording of the non-viewing eye [14].

All recordings took place during the same visit as the VA assessment. MATLABTM, 2026a was used to analyze eye positions. Before analysis, blinks and partial blinks were identified and removed [9,10]. Eye velocity was computed by differentiating position signals and applying a Savitzky–Golay filter for smoothing. Subjects were categorized based on the presence or absence of nystagmus by systematically evaluating FEM traces obtained under binocular and monocular viewing conditions. Nystagmus was defined as repetitive cycles consisting of a fast-phase saccade followed by a slower, prolonged slow phase with linear or decreasing velocity during attempted steady fixation [46].

An automated interface incorporating the Engbert and Kliegl algorithm was used to detect microsaccades (<1°), fixational saccades (>1°), and fast phases of nystagmus [17,18,47]. The amplitude of fast FEMs was computed as the Euclidean norm of the positional displacement:

(1) $$Amplitude=\sqrt{{\left(x_{stop}-x_{start}\right)}^{2}+{\left(y_{stop}-y_{start}\right)}^{2}}$$

Once all fast FEMs, blinks, and artifacts were identified and masked, the remaining continuous epochs were classified as slow FEMs. Slow FEM periods were defined as epochs between fixational saccades or quick phases of nystagmus. We removed 20 milliseconds from the start and end of each of these epochs to exclude periods of acceleration and deceleration of the eye during fast FEMs and blinks [48].

Slow FEM velocity for each intersaccadic interval was calculated as the average magnitude of the instantaneous velocity vector:

(2) $$Velocity_{slow}=\frac{1}{N}\sum_{i=1}^{N}\sqrt{{\left(\frac{dx_{i}}{dt}\right)}^{2}+{\left(\frac{dy_{i}}{dt}\right)}^{2}}$$

These fast FEMs, governed by shared neural circuitry, follow the eye position-velocity main sequence [42,49,50,51,52,53,54,55,56]. Main sequence analysis of pooled FEMs was used to identify and exclude noisy data points [14,48]. Epochs between fixational saccades and fast phases were classified as drifts or slow phases depending on the presence of nystagmus. To separate fast FEM from slow FEMs, we applied velocity threshold first, all fast phases (microsaccades, fixational saccades, and nystagmus fast phases) were isolated, and the remaining continuous segments were classified as slow fixational eye movements. A detailed description of the Engbert velocity-based detection algorithm and its application to identifying fast FEMs is provided in the Supplementary Methods. Supplementary Figure S1 show representative eye position and velocity traces from (i) a control subject, (ii) an amblyopic subject without nystagmus, and (iii) an amblyopic subject with nystagmus, and demonstrate the separation of fast and slow FEMs. We pooled microsaccades, fixational saccades, and nystagmus quick phases as fast fixational eye movements (fast FEMs) and pooled intersaccadic drift and slow phases of nystagmus as slow FEMs.

### 2.3. The Bivariate Contour Ellipse Fixation and Vergence Stability Metric

The bivariate contour ellipse area (BCEA) encompassing 68% of fixation points was used to measure the fixation stability of FE and AE. This was computed as follows:

*BCEA* = *π* x *2σ* x *σy √1 − p2* (3)

where 2.291 is the χ^2^ value (2 degrees of freedom) corresponding to a probability of 0.68; σx and σy are the standard deviations of horizontal (x) and vertical (y) eye positions, respectively; and p is the product–moment correlation between the horizontal and vertical components. Vergence instability was quantified by calculating the difference in horizontal and vertical eye positions between the amblyopic and fellow eye at each time point, using the same BCEA formula. Instead of using raw eye positions, the standard deviations and product–moment correlation of these differences were used to characterize instability. A log_10_ transformation was used to normalize the resulting BCEAs. Because the raw BCEA values were *log_10_* transformed, the scale is monotonic and includes negative values. A mathematically smaller (more negative) logBCEA corresponds to a smaller ellipse area and greater fixation and vergence stability. Conversely, mathematically higher (increasing positive) logBCEA values indicate greater fixation and vergence instabilities.

### 2.4. Quantification of Time-Based Control of Eye Deviation Using DBSCAN

To analyze eye alignment, horizontal and vertical eye position data were first smoothed using a moving average filter to remove fast eye movements and isolate fixational positions. The difference in eye position between the left and right eye was then computed, and composite eye alignment, defined in Equation (4.1), was calculated for each trial and subject. To assess temporal control of alignment, eye deviation data were clustered using a 2D density-based spatial clustering of applications with noise (DBSCAN) algorithm [57,58,59], which identifies stable alignment periods based on data density. Parameters were tuned to exclude rapid transitions while avoiding excessive fragmentation. Clusters were classified based on their mean positional deviation. As a benchmark for normal alignment, clusters with mean positions falling within ±3.5° horizontally and ±2.0° vertically were labeled as well aligned. These thresholds correspond to the 95th percentile range of eye deviation variability observed in healthy control subjects [59]. Clusters with mean deviations outside this threshold window were classified as misaligned. To prevent over-segmentation, clusters with centroids within 1° horizontally and 0.5° vertically were merged into unified alignment states, applying the same criteria to both well-aligned and misaligned clusters. From the final clusters, two metrics were derived: “percentage time of no misalignment,” indicating the percentage of time in each trial where the eye positions were within “well-aligned” state as defined above, and a time-based weighted mean eye deviation that quantifies the average magnitude of eye deviation clusters weighted by the proportion of time spent in each cluster using the following Equations (4.2–4.4):

(4.1) $$Composite=\sqrt{Horizontal^{2}+Vertical^{2}}$$

(4.2) $$FT_{n}=\frac{T_{n}}{T_{total}}$$

(4.3) $$ClusterComposite_{n}=Composite_{n}\times FT_{n}$$

(4.4) $$Mean_{weighted}=\frac{\sum ClusterComposite_{n}}{T_{total}}$$

**Figure 1:**
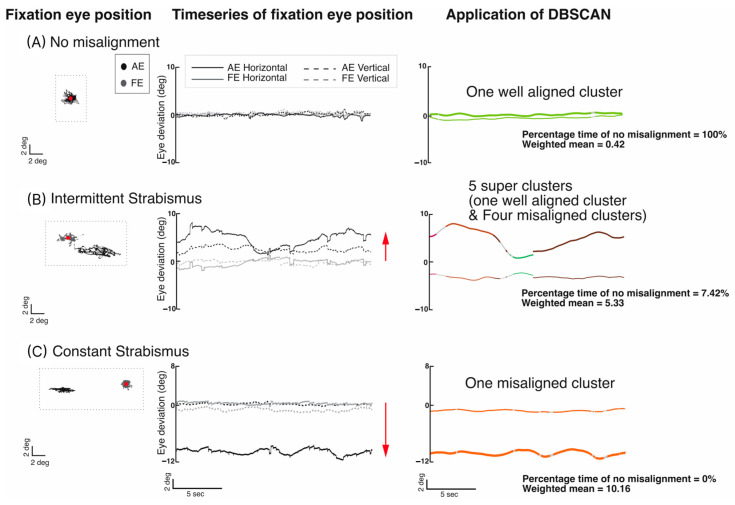
Left panel: Horizontal eye position (x axis) and vertical eye position (y axis) of the fellow/dominant eye (gray) and amblyopic/non-dominant eye (black) from (**A**) a subject with no misalignment, (**B**) a subject with intermittent strabismus where the left eye (gray) is fixing and the right eye (black) shows rightward and downward movement (exodeviation), and (**C**) a subject with constant strabismus where the left eye (gray) is fixing and the right eye (black) shows leftward movement (esodeviation). Red dot = fixing eye. Middle panel: Time series of horizontal and vertical eye positions of fellow/dominant and amblyopic/non-dominant eye from the three subjects. Red arrows = direction of eye deviation. Right panel: Output of DBSCAN analysis. Thick lines = horizontal eye deviation; thin lines = vertical eye deviation over time. Green colors = well-aligned clusters, warm colors = misaligned clusters.

A higher time-weighted mean value reflects a greater degree of sustained eye deviation over extended periods. Figure 1 presents a pipeline for quantifying eye deviation in three subjects—well aligned, intermittent strabismus, and constant strabismus. Panel 1 shows horizontal and vertical eye positions during fixation. Panel 2 illustrates eye movement data of the fellow and amblyopic eye, revealing increasing deviation from subject A to C. Panel 3 displays DBSCAN clustering of eye alignment states: green for well aligned and warmer colors for misaligned. The control subject shows one well-aligned cluster (100% time of no misalignment; weighted mean, 0.42), intermittent strabismus shows mixed clusters (7.42% time of no misalignment; weighted mean, 5.33), and constant strabismus shows only misaligned clusters (0% time of no misalignment; weighted mean, 10.16).

### 2.5. Statistical Analysis

The analysis was performed using Statistical Package for Social Sciences (SPSS) 26. Normality of data was evaluated using the Kolmogorov–Smirnov test. The ages and log stereoacuity of controls, SA, and S groups were compared using one-way ANOVA tests. Two-way mixed-effects model [one within-subject factor: eye (fellow and amblyopic eyes) and one between-subject factor: group [controls vs. strabismus without amblyopia (S) vs. strabismus with amblyopia (SA)] was used to analyze log visual acuity.

To assess group-level differences in FEM metrics, two-way linear mixed-effects models were conducted with Bonferroni-corrected post hoc comparisons. Model assumptions were evaluated by inspection of residual distributions. The dependent variables included FI, fast FEM amplitudes, and slow FEM velocities. The models incorporated one between-participants factor (n = 3)—control vs. S vs. SA—and one within-participants factors—eye_viewing conditions (n = 6) (amblyopic eye in AEV, fellow eye in AEV, fellow eye in FEV, amblyopic eye in FEV, fellow eye in BEV, and amblyopic eye in BEV conditions). For VI, the linear mixed model incorporated one between-participants factor (n = 3)—control vs. S vs. SA—and one within-participants factor—viewing conditions (n = 3) (AEV, FEV, and BEV conditions). Multiple regression analysis was used to determine the factors influencing FI, fast FEM amplitude, slow FEM velocity of fellow/dominant and amblyopic/non-dominant eyes, and VI. The model included age, log-corrected distance VA of fellow/dominant and amblyopic/non-dominant eyes, log stereoacuity, and eye deviation. For all regression models, linearity was assessed by partial regression plots and a plot of studentized residuals against the predicted values. The independence of residuals was assessed by a Durbin–Watson statistic, which was <2.4 for all models. There was homoscedasticity as assessed by visual inspection of a plot of studentized residuals vs. non- standardized predicted values. There was no evidence of multicollinearity as assessed by tolerance values of >0.1 with VIF values of <3. There were no studentized deleted residuals greater than ±3 standard deviations. The frequency of FEM waveforms in SA and S groups and strabismus subtypes (infantile strabismus, accommodative esotropia, exotropia) in SA and S groups were compared using Fisher’s exact test. Non-parametric tests were employed for measures that failed the normality test, including the percentage of time spent in well-aligned states (% time of no misalignment) and time-weighted mean eye deviation. Group differences among controls, SA, and S groups were assessed using one-way Kruskal–Wallis test. Within-subject differences across viewing conditions (BEV, FEV/DEV, AEV/NDEV) were analyzed using the Friedman test. Post hoc comparisons were conducted for all statistically significant results. All statistical tests had a critical alpha (significance) value of 0.05.

## 3. Results

There were no differences in mean age (years) between controls [10 ± 2.2], strabismus with amblyopia (SA) [13 ± 12], and strabismus without amblyopia (S) groups (17 ± 15) [F = 2.3, *p* = 0.1)]. The age range for the SA group was 3–64 years, while the S group ranged from 4 to 57 years. Among the 56 participants with strabismic amblyopia, 24 (42.9%) had infantile strabismus, 22 (39.3%) had accommodative or acquired esotropia, and 10 (17.9%) had intermittent or constant exotropia. In contrast, among the 19 strabismus participants without amblyopia, five (26.3%) had infantile strabismus, four (21.1%) had accommodative or acquired esotropia, and 10 (52.6%) had intermittent or constant exotropia. A significant difference in the distribution of strabismus types was observed between SA and S groups (Fischer exact test; *p* = 0.02). Due to the smaller sample in each subtype, comparisons of FEMs across strabismus subtypes were not performed. A significant two-way interaction in visual acuity was found (F = 18.71, *p* < 0.0001), with significant differences across groups (F = 40.91, *p* < 0.0001), and eyes (F = 27.61, *p* < 0.0001). The mean ± SEM visual acuity of fellow/dominant and amblyopic/non-dominant eye of the three groups across the three viewing conditions was compared (control: DE −0.05 ± 0.04, NDE −0.01 ± 0.04; S: DE −0.01 ± 0.04, NDE 0.04 ± 0.04; SA: FE 0.04 ± 0.02, AE 0.41 ± 0.02). As expected, visual acuity differed significantly between the amblyopic and fellow eyes within the SA group (*p* < 0.0001). In addition to reduced acuity in the amblyopic eye, visual acuity of the fellow eye in the SA group was significantly worse than that of the dominant eye (*p* < 0.05) and non-dominant eye in controls (*p* < 0.05). Among the 75 participants with strabismus, six (five SA and one S) demonstrated gross stereopsis (3500 arcsec) (i.e., positive fly response), while 29 (25 SA and four S) had no measurable stereopsis (i.e., negative fly response). Thus, the SA group showed a higher percentage of absent/gross stereopsis (54%) compared with the S group (28%). In contrast, 45% of participants in the SA group had moderate stereopsis compared with 33% in the S group, and only 2% of SA participants demonstrated fine stereopsis compared with 39% in the S group (χ^2^, *p* = 0.0001). Post hoc analysis revealed a significant difference between the SA and S groups in the fine stereopsis category (χ^2^, *p* = 0.0001).

### 3.1. Fixation Instability (FI) in Strabismus Subjects with and Without Amblyopia

Previous research has established that fixation instability in both strabismus and amblyopia can arise either from the presence of nystagmus or from alterations in physiologic fixation eye movements (FEMs), including increased amplitudes and drift velocities [11,12,14]. We sought to explore how the FI is modulated under different viewing conditions in subjects with strabismic amblyopia and strabismus without amblyopia with and without nystagmus.

**Figure 2:**
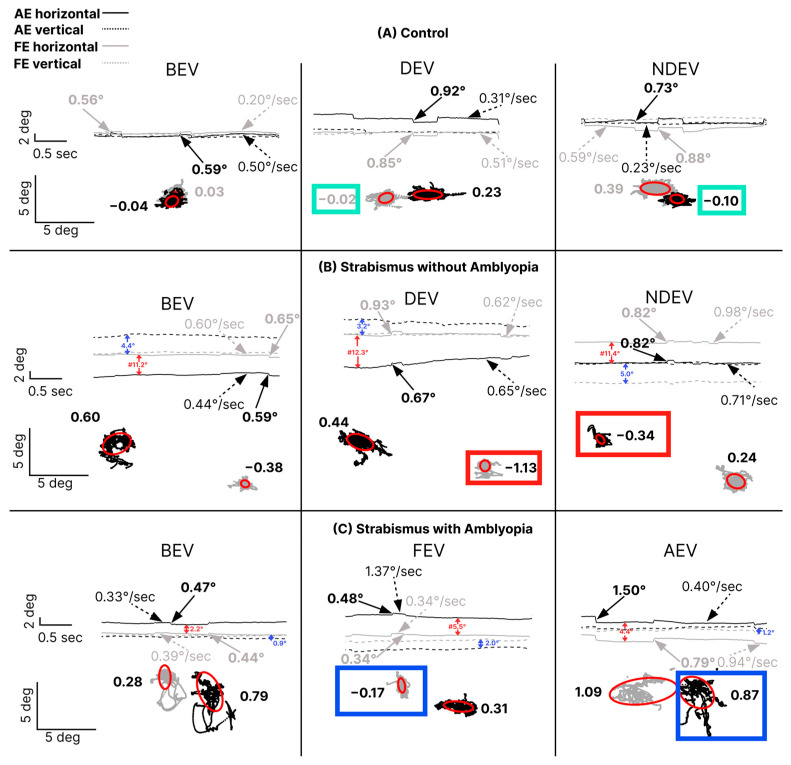
Fixation instability and eye position time series data from a control (**A**), strabismic subject without amblyopia (**B**), and strabismic subject with amblyopia (**C**) under three viewing conditions: binocular viewing (BEV; left column), fellow/dominant-eye viewing (FEV/DEV; middle column), and amblyopic/non-dominant-eye viewing (AEV/NDEV; right column). Time series section (above): The *x*-axis represents time, and the *y*-axis represents horizontal (solid lines) and vertical (dashed lines) eye positions for the fellow/dominant (gray) and amblyopic/non-dominant (black) eyes. Rightward and upward movements correspond to positive horizontal and vertical axes, respectively. Solid arrows represent fast FEM amplitudes (in degrees); dotted arrows represent slow FEM velocities (degrees/second). Numbers in black and gray represent fast FEM amplitude and slow intersaccadic drift velocity of FEM of the amblyopic/non-dominant and fellow/dominant eye, respectively. The horizontal and vertical deviations are denoted in red and blue arrows, respectively. The “#” symbol denotes a break in the *y*-axis scale to accommodate larger ocular misalignments (in degrees) observed in subjects with strabismus. Fixation stability (bottom): Fixation stability is quantified using 68% bivariate contour ellipse area (BCEA; red ellipse), with higher log BCEA values indicating greater instability. Rightward and upward movements correspond to positive horizontal and vertical axes, respectively. The “#” symbol again denotes a break in the *y*-axis scale to accommodate larger ocular misalignments (in degrees). The fixation instability in the viewing eye under monocular viewing is highlighted with a box (green box = control, red box= strabismus without amblyopia and blue box = strabismus amblyopia). In our example, in strabismus without amblyopia where the non-dominant left eye is shifted to the left is indicative of left exotropia, and strabismus with amblyopia where the amblyopic left eye is shifted to the right is indicative of left esotropia.

Figure 2 illustrates time series and FI in the fellow/dominant and amblyopic/non-dominant eyes of three subjects—control (a), strabismic subject without amblyopia (b), and strabismic subject with amblyopia (c)—across three viewing conditions. Larger ellipses with higher log BCEA values indicate greater FI. The subject with amblyopia had greater FI in the amblyopic eye compared to the non-dominant eye of the strabismic subject without amblyopia and the control subject. In addition, there was a greater interocular difference in FI in the amblyopic subject where the FI in the AE in AEV (0.87) was worse than the FI in FE during FEV (−0.17) (panel C, blue boxes) compared with the interocular fixation difference between the non-dominant eye during non-dominant-eye viewing (−0.34) and the dominant eye during dominant-eye viewing (−1.13) in the subject without amblyopia (panel B, red boxes) and control [non-dominant eye during non-dominant-eye viewing (−0.10); dominant eye during dominant-eye viewing (−0.02)] (panel A, green boxes). The time series demonstrated an absence of nystagmus, with higher fixational saccade amplitudes observed in the amblyopic eye during AEV compared to the fellow eye during FEV in the subject with strabismus with amblyopia (panel C).

**Figure 3:**
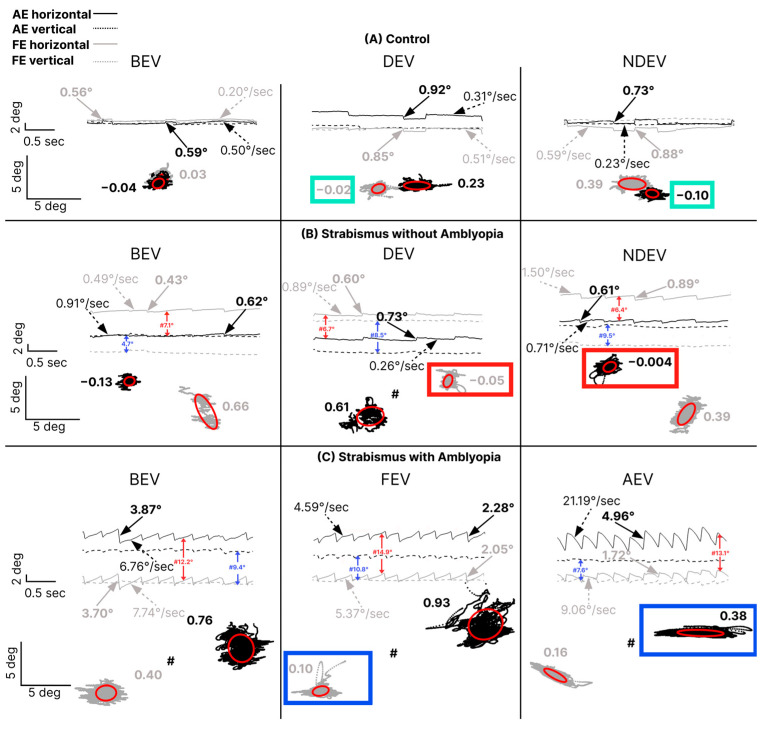
Fixation instability and eye position time series data from a control (**A**), strabismic subject without amblyopia with nystagmus (**B**), and strabismic subject with amblyopia with nystagmus (**C**) under three viewing conditions: both-eye viewing (BEV; left column), fellow/dominant-eye viewing (FEV/DEV; middle column), and amblyopic/non-dominant-eye viewing (AEV/NDEV; right column). Time series section (above): The *x*-axis represents time, and the *y*-axis represents horizontal (solid lines) and vertical (dashed lines) eye positions for the fellow/dominant (gray) and amblyopic/non-dominant (black) eyes. Rightward and upward movements correspond to positive horizontal and vertical axes, respectively. The horizontal and vertical deviations are denoted in red and blue arrows, respectively. Solid arrows represent fast FEM amplitudes (degrees); dotted arrows represent slow FEM velocities (degrees/second). Numbers in black and gray represent fast FEM amplitude and slow intersaccadic drift velocity of FEM of the amblyopic/non-dominant and fellow/dominant eye, respectively. The horizontal and vertical deviations are denoted in red and blue arrows, respectively. The “#” symbol denotes a break in the *y*-axis scale to accommodate larger ocular misalignments (in degrees) observed in subjects with strabismus. Fixation stability (bottom): Fixation stability is quantified using 68% bivariate contour ellipse area (BCEA; red ellipse), with higher log BCEA values indicating greater instability. Rightward and upward movements correspond to positive horizontal and vertical axes, respectively. The “#” symbol again denotes a break in the *y*-axis scale to accommodate larger ocular misalignments (in degrees). The fixation instability in the viewing eye under monocular viewing is highlighted with a box (green box = control, red box= strabismus without amblyopia and blue box = strabismus amblyopia). In our example, strabismus without amblyopia where the non-dominant left eye is shifted to the left is indicative of left exotropia, and strabismus with amblyopia where the amblyopic right eye is shifted to the right is indicative of right exotropia.

To determine whether this pattern persisted in the presence of nystagmus, Figure 3 presents data from control (A), strabismic subjects without amblyopia (B), and strabismic subject with amblyopia (C) across the three viewing conditions. Both strabismic subjects exhibit manifest nystagmus, and similar to the observed pattern for subjects without nystagmus, those with amblyopia had greater FI in the amblyopic eye compared to the non-dominant eye of strabismic subjects without amblyopia and control subjects. In addition, there was a greater interocular difference in FI in amblyopic subjects where the FI in the AE in AEV (0.38) was worse than the FI in FE during FEV (0.10) (panel C, blue boxes) compared with the interocular fixation difference between the non-dominant eye during non-dominant-eye viewing (−0.004) and the dominant eye during dominant-eye viewing (−0.05) in the subject without amblyopia (panel B, red boxes) and control [non-dominant eye during non-dominant-eye viewing (−0.10); dominant eye during dominant-eye viewing (−0.02), (panel A, green boxes)]. The time series demonstrates the presence of nystagmus, with higher amplitudes of quick phases observed in the amblyopic eye during AEV compared to fellow eye during FEV in strabismic amblyopia subject. Thus, an interocular difference in FI was observed in the viewing eye under monocular viewing in strabismic amblyopia subjects regardless of the presence or absence of nystagmus.

We evaluated FEM waveforms across the SA and S groups and found a significantly higher prevalence of nystagmus in the SA group (nystagmus absent: 29%; nystagmus present: 71%) compared with the S group (nystagmus absent: 63%; nystagmus present: 37%). Chi-square results revealed a significant difference in the frequency of nystagmus between SA and S groups (χ^2^, *p* = 0.007).

Amblyopia and strabismus—regardless of the presence of nystagmus—have consistently been associated with abnormalities in FI [11,12,14]. Because the SA and S groups included only a small number of participants with and without nystagmus, separate analyses of FI based on nystagmus status were not feasible. In addition, global measures such as BCEA lack the sensitivity to reliably identify nystagmus or to characterize abnormalities in drift and slow-phase velocities during fixation. Therefore, in addition to FI, we examined fast and slow FEMs across the three groups—controls, SA, and S—under all viewing conditions and evaluated the visual and oculomotor factors that contribute to these metrics.

### 3.2. FI and Factors Influencing These Metrics

**Figure 4:**
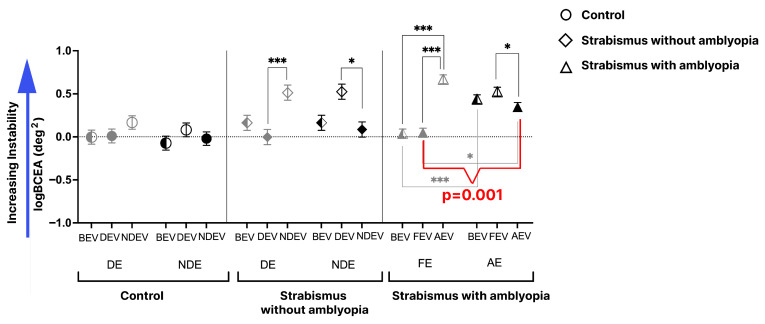
Mean and SEM of fixation instability (FI) or across various groups. The data is categorized per fellow eye (gray) and amblyopic eye (black) across three viewing conditions: both-eye viewing (BEV), fellow/dominant-eye viewing (FEV/DEV), and amblyopic/non-dominant-eye viewing (AEV/NDEV). In monocular viewing conditions, non-viewing eyes are represented as open symbols, and viewing eyes are represented as solid symbols. Black brackets = significant differences within groups; gray brackets = significant difference between fellow- and amblyopic viewing-eye FEMs (i.e., amblyopic eye in AEV, and fellow eye in FEV) (* *p* < 0.05, *** *p* < 0.0001).

Figure 4 plots the cumulative FI data of fellow/dominant and amblyopic/non-dominant eye in the three groups across the three viewing conditions. A two-way interaction revealed significant main effects for group (F = 33.57, *p* < 0.0001) and viewing condition (F = 15.97, *p* < 0.0001), with a significant interaction among viewing condition and group (F = 3.46, *p* < 0.0001). Post hoc comparisons showed that during BEV, the FI of the amblyopic eye in the SA group was higher than the control (*p* < 0.0001). During FEV/DEV and AEV/NDEV, non-viewing-eye instability was significantly elevated in SA and S groups compared to controls (*p* < 0.0001). Post hoc comparison also showed that the viewing-eye FI of the amblyopic eye in AEV was highest in the SA group in AEV compared to the non-dominant eye during NDEV in controls (*p* = 0.001) and S groups (*p* = 0.03). No such differences in the FI of the non-dominant eye in NDEV were seen between controls and S groups (*p* = 1.0). In the SA group, the FI in the amblyopic eye during AEV was significantly higher than in the fellow eye during FEV, a pattern not observed in the S group. In other words, the SA group showed a markedly larger interocular FI difference, with FI increasing from 0.05 ± 0.05 in FEV to 0.35 ± 0.05 in AEV (*p* = 0.001), as indicated by the red brackets.

To examine clinical and demographic influences on FI across viewing conditions, a multiple regression analysis was conducted using subject age, corrected distance visual acuity of both eyes, stereoacuity, and eye deviation (Table 1). Poorer visual acuity correlated with increased viewing-eye FI in the amblyopic/non-dominant eye during AEV/DEV and in the fellow/dominant eye during FEV/DEV. Greater eye deviation was associated with elevated non-viewing-eye FI in the fellow/dominant eye during AEV/NDEV and in the amblyopic/non-dominant eye during FEV/DEV. Additionally, worse stereoacuity predicted increased FI in the amblyopic/non-dominant eye during BEV. These results reinforce that both sensory deficits and motor misalignment contribute to FI, likely through a common underlying visuomotor control mechanism with varying influences from sensory and motor inputs. In this framework, strabismus appears to drive the non-viewing-eye FI, while amblyopia exacerbates the viewing-eye FI—highlighting differential weighting of shared visuomotor control mechanisms in the SA and S groups.

### 3.3. Fast and Slow FEMs and Factors Influencing These Metrics

**Figure 5:**
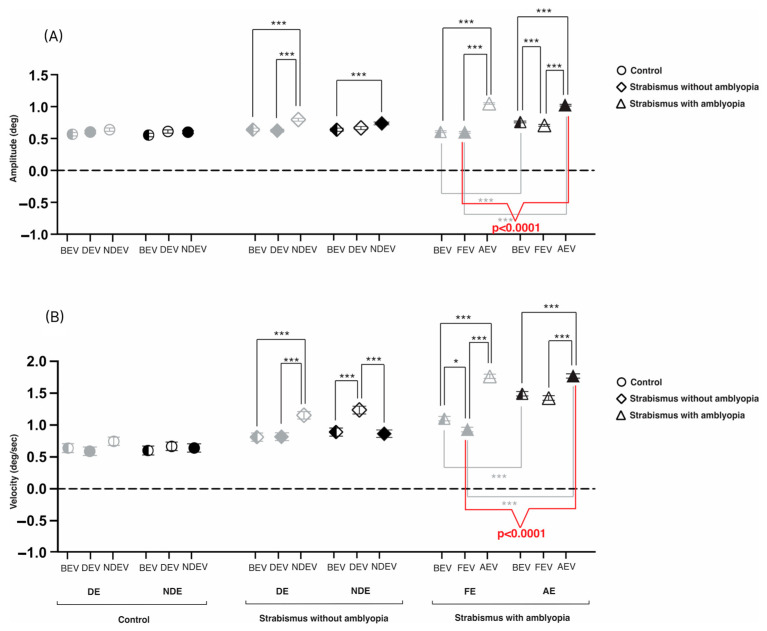
Mean and SEM of (**A**) fast FEM (fixation eye movements) amplitude (degrees) and (**B**) slow FEM velocity (degree/sec) across various groups. The data is categorized per fellow/dominant eye (gray) and amblyopic/non-dominant eye (black) across three viewing conditions: both-eye viewing (BEV), fellow/dominant-eye viewing (FEV/DEV), and amblyopic/non-dominant-eye viewing (AEV/NDEV). In monocular viewing conditions, non-viewing eyes are represented as open symbols, and viewing eyes are represented as solid symbols. Black brackets = significant differences of the same eye across different viewing conditions; gray brackets = significant difference between fellow-eye- and amblyopic-viewing-eye FEMs (i.e., amblyopic eye in AEV and fellow eye in FEV) (* *p* < 0.05, *** *p* < 0.0001).

Figure 5A illustrates the cumulative data of composite amplitudes of fast FEMs across fellow/dominant and amblyopic/non-dominant eyes in control, S, and SA groups across the three viewing conditions. A significant two-way interaction was observed (F = 26.53, *p* < 0.001), with main effects for group (F = 146.09, *p* < 0.0001), and eye_viewing conditions (F = 56.50, *p* < 0.0001). During BEV, amblyopic-eye amplitudes were significantly higher in SA compared to S and controls (*p* < 0.0001), while no differences were found between S and controls. In FEV/DEV, non-viewing-eye amplitudes were elevated in SA versus controls, with no differences in viewing-eye amplitudes. During AEV/NDEV, both non-viewing-eye and viewing-eye amplitudes were highest in SA, followed by S and then controls (*p* < 0.0001). SA also showed significantly greater viewing-eye amplitudes than the S group (*p* < 0.0001). Within the SA group, the interocular differences were more pronounced; i.e., the amblyopic eye in AEV had greater amplitudes than the fellow eye in FEV (amblyopic eye in AEV: 1.02 ± 0.01, fellow eye in FEV: 0.60 ± 0.01; *p* < 0.0001), as indicated by the red brackets.

Figure 5B presents slow FEM velocities across the same groups and conditions. A significant two-way interaction was found (F = 15.13, *p* < 0.0001), with significant differences across groups (F = 356.77, *p* < 0.0001) and eye_viewing conditions (F = 26.87, *p* < 0.0001). During BEV, both eyes in SA showed the highest velocities, followed by S and controls (*p* < 0.0001), while only the non-dominant eye was elevated in S compared to controls. In FEV/DEV and AEV/NDEV, non-viewing-eye velocities of both eyes were significantly higher in SA and S compared to controls (*p* < 0.001), with SA exceeding the S group (*p* < 0.05). Viewing-eye velocities during AEV/NDEV were also significantly elevated in SA and S versus controls (*p* < 0.05), with SA showing higher velocities than S (*p* < 0.0001). Notably, similar to amplitudes in the SA group, amblyopic-eye velocities were significantly higher in AEV (1.77 ± 0.04) than the fellow eye in FEV (0.93 ± 0.04) (*p* < 0.0001), as indicated by the red brackets.

Additionally, we investigated the influence of key clinical and demographic factors, including age, corrected distance visual acuity in both eyes, stereoacuity, and eye deviation, to better understand the underlying contributors to abnormalities of fast FEM amplitudes (Table 2) and slow FEM velocities (Table 3). Age analysis showed that older subjects had greater fast FEM amplitude in non-viewing-eye and viewing-eye conditions in AEV. Viewing- and non-viewing-eye amplitudes of the amblyopic/non-dominant eye are directly related to the visual acuity of the amblyopic/non-dominant eye. The visual acuity of the fellow/dominant eye affects the amplitudes of the fellow/dominant and amblyopic/non-dominant eye in all three viewing conditions. The amplitudes of the amblyopic/non-dominant eye in BEV were higher in those with greater eye deviation. Slow FEM velocities of both eyes are greater in older subjects in both viewing- and non-viewing-eye conditions. Increased visual acuity deficit of the fellow/dominant eye was associated with greater viewing- and non-viewing-eye slow FEM velocities of both eyes. Velocities of both eyes during BEV and FEV/DEV are increased in those with greater eye deviation.

### 3.4. Vergence Instability (VI) and Factors Influencing VI

**Figure 6:**
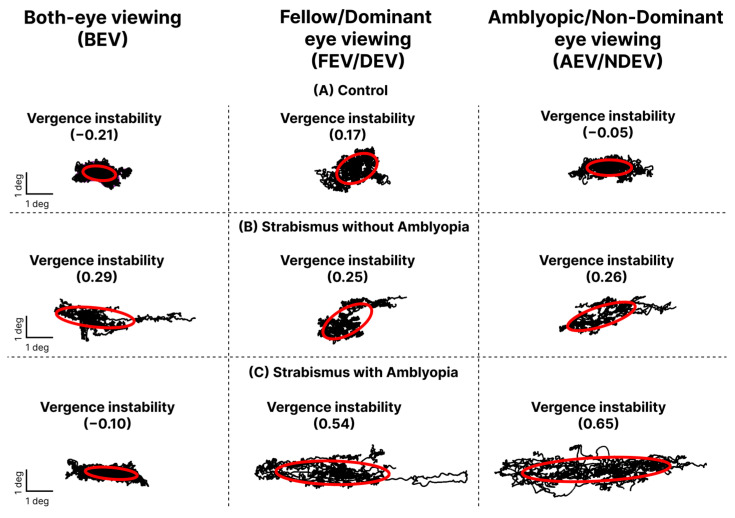
Vergence stability of (**A**) a control subject, (**B**) a subject with strabismus without amblyopia, and (**C**) a subject with strabismus and amblyopia recorded during a 45 s visual fixation trial in primary gaze. Data are shown for both-eye viewing (BEV, left column), fellow/dominant-eye viewing (FEV/DEV, middle column), and amblyopic/non-dominant-eye viewing (AEV/NDEV, right column). Vergence stability is quantified using 68% bivariate contour ellipse area (BCEA) (red ellipse), with higher log BCEA values indicating greater instability.

**Figure 7:**
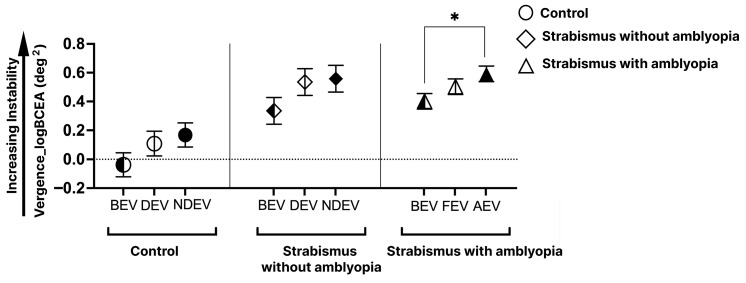
Mean and SEM of vergence instability (VI) per various groups across three viewing conditions: both-eye viewing (BEV), fellow/dominant-eye viewing (FEV/DEV), and amblyopic/non-dominant-eye viewing (AEV/NDEV). Black bracket = significant differences within groups (* *p* < 0.05).

Figure 6 depicts vergence stability (red ellipses) in a control subject, SA, and S subject across three viewing conditions. Larger ellipses with higher log BCEA values indicate greater VI. The SA and S subjects had larger BCEA than the control subject, indicating greater VI under all three viewing conditions. Figure 7 plots the cumulative data of VI in all three groups across the three viewing conditions. A significant main effect for groups (F = 28.01, *p* < 0.0001) and viewing conditions (F = 5.55, *p* = 0.004) with no interaction was observed between groups and viewing conditions (F = 0.12, *p* = 0.98). Significantly worse VI is observed in SA and S groups than controls in all three viewing conditions (*p* < 0.01). Further, in the SA group, the greatest VI was observed in AEV (panel c), with no differences observed across viewing conditions in controls (panel a) and S groups (panel b). Multiple regression analysis revealed greater eye deviation was associated with increased VI in all three viewing conditions (Table 4).

### 3.5. Eye Alignment

**Figure 8:**
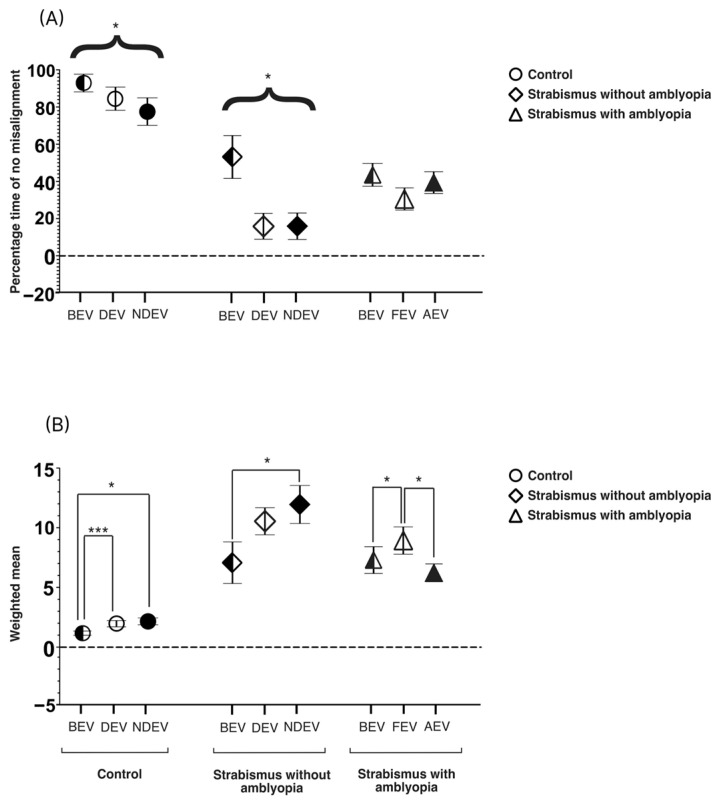
Mean and SEM of (**A**) % time of no misalignment (total percentage of time when both eyes were well aligned) and (**B**) time-based weighted mean per various groups across three viewing conditions: both-eye viewing (BEV), fellow/dominant-eye viewing (FEV/DEV), and amblyopic/non-dominant-eye viewing (AEV/NDEV). Black brackets = significant differences within groups (* *p* < 0.05, *** *p* < 0.0001). 9A showed within group differences (curly brackets) in control (*p* = 0.02) and strabismus without amblyopia (*p* = 0.0006).

As VI, which is driven by eye deviation, showed differences across viewing conditions in the SA group, we examined whether eye alignment changed across viewing conditions. Thus, we computed the “percentage time of no misalignment” when the eyes were well aligned (Figure 8A) across all three groups and viewing conditions. Between-group analyses showed significant difference in BEV [χ^2^(3) = 22.99, *p* < 0.0001], FEV/DEV [χ2(3) = 32.86, *p* < 0.0001] and AEV/NDEV [χ2(3) = 28.87, *p* < 0.0001]. As expected, controls had significantly higher percentage time of no misalignment than SA and S groups across all viewing conditions (*p* < 0.05). Interestingly in AEV/NDEV, SA also showed a higher percentage time of no misalignment than S (*p* = 0.04). Within-group comparisons revealed significant differences, with reduced percentage time of no misalignment in DEV and NDEV than BEV in the control and S groups (*p* < 0.05) and with no such differences observed in FEV and AEV in the SA group.

We also computed time-based weighted means to capture variability in eye deviation across all three groups and viewing conditions (Figure 8B). Between-group analyses revealed significant differences across groups in BEV [χ2(3) = 23.34, *p* < 0.0001], FEV/DEV [χ2(3) = 9.72, *p* = 0.01], and AEV/NDEV [χ2(3) = 27.25, *p* < 0.0001]. As expected, both SA and S had significantly higher weighted means than controls (*p* < 0.05). Interestingly, the S group had a significantly higher weighted mean than SA in NDEV/AEV (*p* = 0.01). Within-group comparisons revealed that controls had the lowest weighted mean in BEV compared with DEV and NDEV (*p* < 0.05). Similarly, S had the lowest weighted mean in BEV when compared with DEV and NDEV (*p* < 0.05). On the other hand, for the SA group, the lowest weighed mean was observed in AEV. Further, in the SA group, FEV showed a significantly higher weighted mean than AEV (*p* < 0.05), showing incomitant eye deviation under monocular viewing. In the S group and controls, no differences were observed between DEV and NDEV (*p* = 0.04).

## 4. Discussion

This study identifies distinct oculomotor markers that differentiate strabismic subjects with amblyopia (SA) and without amblyopia (S) and demonstrates how visual input influences fixation and vergence stability and eye alignment. The main findings were that (1) fixation instability (FI) was greater in the amblyopic eye during AEV than in the fellow eye during FEV in the SA group, whereas no such interocular differences were observed between the nondominant and dominant eyes in the S group. (2) Non-viewing-eye FI was worse in SA and S groups relative to controls. (3) Regression models revealed that visual acuity is a key contributor to increased viewing-eye FI, while eye deviation primarily drives non-viewing-eye FI in both amblyopic and fellow eyes. (4) Vergence instability (VI) is increased in SA and S groups compared to controls and worsens with increasing eye deviation. (5) SA subjects showed the highest fast FEM amplitudes and slow FEM velocities, followed by the S group, with controls exhibiting the lowest values. Moreover, amplitudes and velocities increased more in the AEV than in the FEV within the SA group, suggesting that the observed interocular differences are more strongly driven by coexisting amblyopia than by strabismus alone. (6) Regression analysis identified that distance visual acuity of both the amblyopic and fellow eyes contributes to increased fast-phase FEM amplitudes in both non-viewing- and viewing-eye conditions, while the fellow-eye acuity and eye deviation are key drivers of elevated slow FEM velocities, especially during BEV. (7) Controls and the S groups showed the least eye deviation during BEV, while the SA group exhibited the least deviation during AEV. The SA group also showed incomitance, with significantly greater deviation in FEV than AEV—a pattern absent in the S group.

Vision is fundamental for fine-tuning eye movements, and individuals with low vision—such as those with age-related macular degeneration—often exhibit abnormal FEMs [60,61,62,63]. Previous studies have documented increased FI in amblyopic subjects, with or without strabismus, and linked FI primarily to reduced visual acuity [7,9,48,64]. However, only a few investigations have examined fast and slow FEM abnormalities in these cohorts [10,11,12], and none have systematically evaluated how visual acuity of the amblyopic and fellow eyes, age, stereoacuity, and strabismus angle influence these metrics under different viewing conditions. For the first time, in the current study, we demonstrate that FI is differentially influenced by visual acuity and eye deviation depending on viewing condition: FI in the viewing eye is primarily associated with amblyopic-eye visual acuity, whereas FI in the non-viewing eye is linked to eye deviation. While both factors contribute to FI, fast FEM amplitudes under monocular viewing were associated only with visual acuity deficits, not eye deviation—highlighting the tight correlation between the amplitude of fast FEMs and visual acuity. On the other hand, we found that, similar to FI, slow FEM velocity is influenced by visual acuity, as well as by eye deviation. Our analyses reveal that the relationship between visual acuity and fast FEM amplitude is observed not only for the amblyopic eye but also for the fellow eye. These results highlight the strong interdependence between visual acuity and FEMs across eyes.

Vergence instability (VI) also varied across viewing conditions. Controls exhibited the greatest stability under BEV, with mild reductions under monocular viewing likely due to small increases in deviation. Subjects with strabismus—regardless of amblyopia—showed markedly increased VI. During BEV and FEV, amblyopic subjects fixated with the fellow eye, resulting in greater deviation of the amblyopic eye. Under AEV, better control of the fellow eye reduced deviation and incomitance, highlighting the critical role of visual input in alignment control.

In addition to the above variables, age emerged as a secondary factor influencing FEMs. Consistent with prior reports, younger subjects exhibited greater FI, while older individuals showed increased intersaccadic drift. In our cohort, older age correlated with higher slow-phase velocities in both eyes. However, because age did not differ significantly across groups and interocular differences were observed only in strabismus-with-amblyopia subjects, age is unlikely to be a primary confounder.

Emerging evidence highlights a complex, bidirectional relationship between visual sensory processing and eye movements, particularly in the context of amblyopia and strabismus. Nystagmus and stereopsis are closely linked, with the presence of nystagmus often indicating disrupted binocular development and predicting poor recovery of stereopsis following treatment [65]. Mechanistic insights from animal studies reveal that the earliest structural and functional deficits in amblyopia originate in the primary visual cortex [V1), where abnormal input affects downstream oculomotor control [66,67]. These disruptions are especially consequential during the critical period of stereopsis development in early infancy, when the visual system is highly sensitive to discordant binocular signals [4,68]. In strabismic amblyopia, early abnormal binocular experience leads to maldevelopment in cortical and subcortical pathways—including V1, MT, and MST—resulting in fixation instability and fusion maldevelopment nystagmus [4,69]. In contrast, individuals with strabismus but without amblyopia are more likely to have experienced normal binocular development in early life, making them less susceptible to nystagmus and more likely to retain or recover stereopsis. This distinction may explain the higher prevalence of nystagmus and poor stereoacuity in strabismic individuals with amblyopia. Overall, these neurodevelopmental changes result in deficits in visual acuity, contrast sensitivity, and binocular integration, with compromised fidelity of visual input from V1 directly impairing the motor output required for stable fixation.

In strabismus, animal models reveal that FI also arises from abnormal development of vergence eye movements [70,71]. The superior colliculus, a central hub for saccade generation and vergence control, plays a pivotal role [50,51,55]; stimulation of its rostral pole can modify the strabismic angle, likely via its influence on vergence neurons. Computational models further suggest that microsaccade timing and execution are regulated by reciprocal inhibition between omnipause and long-lead burst neurons [56,72]. Disruption of this balance contributes to increased fixation scatter and ocular misalignment in strabismic individuals.

While the relatively small sample size limited detailed waveform analyses across subtypes and introduced some variability due to the inclusion of both constant and intermittent strabismic subjects, these factors did not significantly affect viewing-eye FEM outcomes. A key limitation of our study is that ocular dominance was defined solely based on the eye with superior visual acuity. While this operational approach was practical for our analysis—given prior studies demonstrating a link between fixation eye movements and acuity performance—it is important to recognize that ocular dominance is a multifactorial phenomenon that cannot be fully characterized by acuity alone. Previous research has shown that dominance influences various aspects of visual perception, including binocular rivalry, stereopsis, and visual acuity, and that multiple assessment techniques are recommended for accurate classification [73,74,75]. Future studies should incorporate multiple tests of ocular dominance—such as sighting dominance, sensory dominance, and binocular rivalry measures—to provide a more comprehensive characterization. Additionally, examining the relationship between ocular dominance and FEMs using these complementary methods will help clarify whether dominance exerts a measurable influence on fixation stability in strabismic subjects without amblyopia. Further, although the groups were not perfectly age-matched, prior work indicates that age does not significantly influence interocular FEMs [76], making it unlikely that minor age differences contributed to the observed FEM patterns. Another limitation arises from the varying subtypes of strabismus across strabismic subjects with and without amblyopia. As the majority of strabismic amblyopia subjects either had infantile strabismus or accommodative esotropia, whereas those with strabismus without amblyopia were more likely to have exotropia, the sample size within individual strabismus subgroups was insufficient to permit meaningful comparisons of fixational eye movements across specific diagnoses (e.g., intermittent exotropia, accommodative esotropia). Future studies with larger cohorts will be important to systematically examine subtype-specific differences in fixational eye movements and to determine how fixation instability may vary across distinct forms of strabismus.

In summary, this study highlights robust mechanistic links between visual input and oculomotor control, demonstrating that fixation instability, vergence instability, and eye deviation are differentially modulated in strabismic individuals with and without amblyopia. The strong association between visual acuity and FEM metrics suggests that eye-tracking-based measures may serve as objective indicators of visual function in strabismus and amblyopia, although these relationships must be interpreted in the context of the frequent co-occurrence of these conditions. Notably, interocular differences in FEMs were pronounced only in strabismic individuals with amblyopia. As portable, high-resolution eye-tracking technologies and AI (artificial intelligence)-based analytic tools continue to advance, integrating such metrics with normative and pathological fixation-instability datasets may support earlier and more accurate amblyopia detection—particularly in young children who cannot reliably complete standard acuity assessments. Additionally, the differential effects of viewing conditions on vergence instability and eye deviation underscore the value of evaluating both binocular and monocular states when considering interventions such as occlusion therapy, binocular training, or surgery. Taken together, the distinct oculomotor signatures identified here provide new insight into sensory–motor integration deficits in strabismus and amblyopia and lay important groundwork for diagnostic innovation. Future studies should extend this work to younger populations, including infants and toddlers, to characterize the developmental trajectory of FEMs and identify early oculomotor markers predictive of amblyopia risk and treatment outcomes.

## Figures and Tables

**Table 1:** Multiple regression model of fellow-eye and amblyopic-eye fixation instability in controls, strabismus with amblyopia, and strabismus without amblyopia under different viewing conditions.

Separate Fellow-Eye and Amblyopic-Eye Fixation Instability			
Variables	**FI of FE in BEV** **[FI of AE in BEV]**	**FI of FE in FEV** **[FI of AE in FEV]**	**FI of FE in AEV** **[FI of AE in AEV]**
Age	−0.24 [−0.15]	**−0.24** [−0.09]	0.06 **[−0.004]**
AE visual acuity	−0.15 [0.21]	**−0.07** [0.20]	0.24 * [0.51] ***
FE visual acuity	0.09 [0.01]	**0.23** [0.01]	−0.002 **[0.05]**
Log stereoacuity	0.11 [0.27] *	**0.04** [0.09]	0.12 **[−0.07]**
Eye deviation	0.18 [0.17]	**0.01** [0.37] ***	0.26 * [0.16]
R^2^, F	0.09, 1.75 [0.20, 4.27] *	**0.11, 2.08** [0.21, 4.63] *	0.20, 4.37 * [0.28, 6.88] ***

AE = amblyopic eye; FE = fellow eye; FEV = fellow-eye viewing; AEV = amblyopic-eye viewing; BEV = both-eye viewing; FI = fixation instability. The top row in each cell in the individual eye position FI section represents data of the FE, whereas the data in square brackets [] represent values of the AE. * *p* < 0.05; *** *p* < 0.001. Values in bold indicate viewing-eye results; i.e., FE in FEV, and AE in AEV.

**Table 2:** Multiple regression model of fellow-eye and amblyopic-eye amplitudes of fast FEM in controls, strabismus with amblyopia, and strabismus without amblyopia under different viewing conditions.

Separate Fellow-Eye and Amblyopic-Eye Fast-Phase FEM Amplitudes			
Variables	**Fast Phase of FE in BEV** **[Fast Phase of AE in BEV]**	**Fast Phase of FE in FEV** **[Fast Phase of AE in FEV]**	**Fast Phase of FE in AEV** **[Fast Phase of AE in AEV]**
Age	−0.07 [−0.09]	**−0.13** [−0.06]	0.24 * [0.26] *
AE visual acuity	−0.16 [−0.13]	**−0.18** [−0.26] *	0.18 [0.21] *
FE visual acuity	0.35 * [0.30] *	0.37 * [0.34] *	0.35 * [0.43] ***
Log stereoacuity	0.04 [0.03]	**−0.09** [0.07]	0.04 **[−0.02]**
Eye deviation	0.19 [0.35] *	**0.09** [0.16]	−0.02 **[0.06]**
R^2^, F	0.17, 3.38 * [0.22, 4.85] *	0.17, 3.55 * [0.17, 3.41] *	0.26, 5.93 *** [0.35, 9.16] ***

AE = amblyopic eye; FE = fellow eye; FEV = fellow-eye viewing; AEV = amblyopic-eye viewing; BEV = both-eye viewing; FEM = fixation eye movements. The top row in each cell in the individual eye position: top section represents data of the FE, whereas the data in square brackets [] represent values of the AE. * *p* < 0.05; *** *p* < 0.001. Values in bold indicate viewing-eye results; i.e., FE in FEV and AE in AEV.

**Table 3:** Multiple regression model of fellow-eye and amblyopic-eye velocities of slow FEM in controls, strabismus with amblyopia, and strabismus without amblyopia under different viewing conditions.

Separate Fellow-Eye and Amblyopic-Eye Slow-Phase FEM Velocities			
Variables	**Slow Phase of FE in BEV** **[Slow Phase of AE in BEV]**	**Slow Phase of FE in FEV** **[Slow Phase of AE in FEV]**	**Slow Phase of FE in AEV** **[Slow Phase of AE in AEV]**
Age	−0.04 [0.09]	0.40 *** [0.31] *	0.37 *** [0.34] ***
AE visual acuity	−0.10 [−0.10]	**−0.08** [−0.07]	−0.11 **[−0.01]**
FE visual acuity	0.19 [0.34] *	0.57 *** [0.42] ***	0.52 * [0.51] ***
Log stereoacuity	0.09 [−0.17]	**0.01** [0.08]	0.12 **[0.13]**
Eye deviation	0.32 * [0.40] ***	0.18 * [0.29] *	0.12 **[1.77]**
R^2^, F	0.17, 3.34 * [0.27, 6.32] ***	0.53, 19.04 *** [0.43, 12.40] ***	0.48, 15.37 *** [0.42, 12.21] ***

AE = amblyopic eye; FE = fellow eye; FEV = fellow-eye viewing; AEV = amblyopic-eye viewing; BEV = both-eye viewing; FEM = fixation eye movements. The top row in each cell in the individual eye position: top section represents data of the FE, whereas the data in square brackets [] represent values of the AE. * *p* < 0.05; *** *p* < 0.001. Values in bold indicate viewing-eye results; i.e., FE in FEV and AE in AEV.

**Table 4:** Multiple regression model evaluating vergence stability in controls, strabismus with amblyopia, and strabismus without amblyopia under different viewing conditions.

Variables	VI of FE in BEV	VI of AE in BEV	VI of BE in BEV
Age	−0.06	0.07	−0.07
AE visual acuity	0.12	0.14	0.14
FE visual acuity	−0.01	0.02	−0.10
Log stereoacuity	0.15	0.09	0.23
Eye deviation	0.37 ***	0.37 ***	0.31 *
R^2^, F	0.21, 4.53 *	0.22, 4.84 *	0.21, 4.60 *

AE = amblyopic eye; FE = fellow eye; FEV = fellow-eye viewing; AEV = amblyopic-eye viewing; BEV = both-eye viewing; VI = vergence instability. The top row in each cell in the individual eye position FI section represents data of the FE, whereas the data in square brackets [] represent values of the AE. * *p* < 0.05; *** *p* < 0.001.

## Data Availability

The data supporting the findings of this study contain patient-level information and cannot be made publicly available due to privacy and ethical restrictions. De-identified data may be shared upon reasonable request to the corresponding author and subject to institutional review and data use agreements, in compliance with applicable regulations and ethical guidelines.

## Supplementary Materials

The following supporting information can be downloaded at: https://www.mdpi.com/article/10.3390/jemr19030047/s1, Figure S1: epoch of right and left eye horizontal and vertical positions (solid lines, left y-axis) and their corresponding horizontal and vertical velocities (dotted lines, right y-axis).

## Abbreviations

The following abbreviations are used in this manuscript:

FI	Fixation instability
VI	Vergence instability
FEM	Fixation eye movement
SA	Strabismus with amblyopia
S	Strabismus without amblyopia
C	Control
BEV	Both-eye viewing
AEV	Amblyopic-eye viewing
FEV	Fellow-eye viewing
DEV	Dominant-eye viewing
NDEV	Nondominant-eye viewing
AE	Amblyopic eye
FE	Fellow eye
