# Supplementary material for: Differential Fixation and Eye Alignment Patterns in Strabismus with and Without Amblyopia Across Viewing Conditions
Source: J Eye Mov Res. 2026 May 3;19(3):47. doi: 10.3390/jemr19030047 (PMC13214692; doi:10.3390/jemr19030047)
Supplement: Supplementary file 1 [file jemr-19-00047-s001.zip › jemr-4167759-supplementary.pdf]

Engbert Velocity-Based Saccade Detection Algorithm (Explanation for Figure)

Saccadic events were detected using the velocity-based algorithm originally described by **Engbert and Kliegl (2003)**, which identifies saccades as transient excursions in eye velocity relative to the noise characteristics of the signal rather than using absolute velocity thresholds.

Step 1: Velocity Estimation from Eye Position

Horizontal and vertical eye position signals were first converted to velocity using a five-point moving average derivative. The instantaneous velocity v_n at sample n was computed using the following equation:

$$v_n = \frac{f_s}{6}[(x_{n+2} + x_{n+1}) - (x_{n-1} + x_{n-2})]$$

This method is an important differentiation step to calculate the eye velocity while simultaneously smoothing the data, which significantly suppress high-frequency noise prior to threshold detection.

Step 2: Robust Estimation of Velocity Noise

To account for inter-individual differences in noise and sampling variability, velocity thresholds were defined relative to the data itself. The standard deviation of the velocity noise (σ) for each dimension was estimated using a robust, median-based estimator:

$$\sigma_v = \sqrt{\text{median}(v^2) - \big(\text{median}(v)\big)^2}$$

This estimator is mathematically less sensitive to the extreme outliers introduced by the saccades themselves, ensuring that detection is driven by the underlying signal-to-noise characteristics rather than absolute velocity peaks.

Step 3: Elliptical Velocity Threshold

A data sample was classified as part of a potential saccadic event when its two-dimensional velocity vector exceeded an elliptical threshold defined by:

$$\left(\frac{v_x}{\lambda\sigma_x}\right)^2 + \left(\frac{v_y}{\lambda\sigma_y}\right)^2 > 1$$

where σ_x and σ_y ere are the noise estimates for horizontal and vertical velocity, and λ is a dimensionless scaling factor set to 5 (λ = 5). This criterion requires simultaneous high velocity relative to noise in at least one direction, which significantly reduces false detections due to isolated noise spikes.

In the Supplemental Figure 1, the velocity threshold crossings correspond to the transient peaks in the velocity trace.

Step 4: Temporal Clustering and Event Definition

Consecutive samples exceeding the elliptical velocity threshold were grouped into a single saccadic event if they occurred contiguously in time. To avoid spurious detections driven by transient noise, these events were required to exceed a minimum duration of 6 consecutive samples (corresponding to 12ms at the 500Hz sampling rate). Furthermore, to be classified as a true physiological saccade, the algorithm required temporal overlap between the left and right eyes (binocular saccade verification).

Step 5: Mapping Back to Position Space

Detected saccades were then projected back onto the temporal eye position traces to verify that each event corresponded to a rapid, step-like spatial displacement. As illustrated in the figure, the algorithmically flagged velocity bursts consistently coincide with abrupt position shifts, demonstrating strong agreement between kinematic detection and position-level behavior.

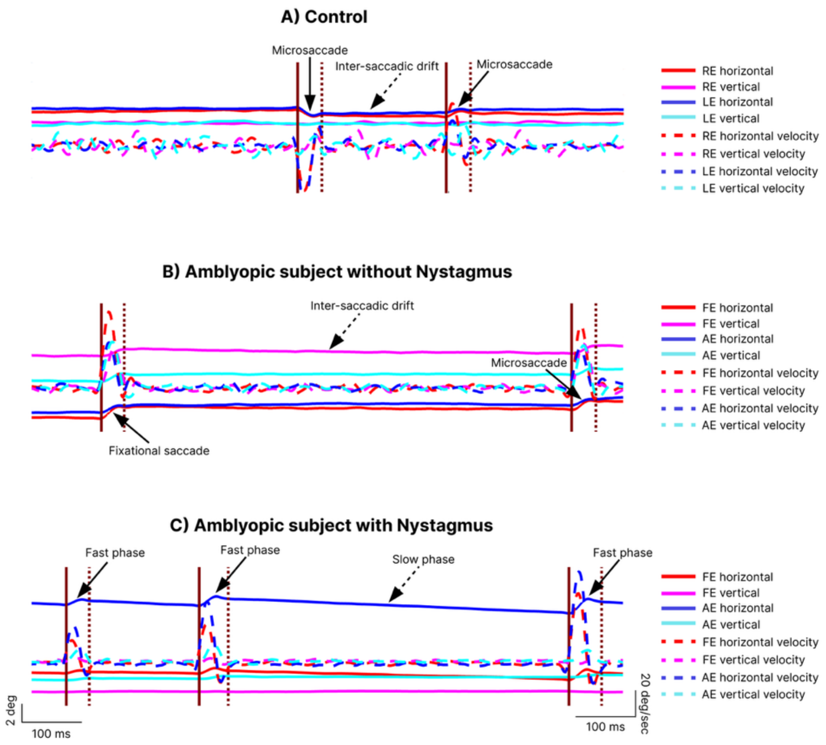

Supplemental Figure 1 caption:

The figure represents an epoch of right and left eye horizontal and vertical positions (solid lines, left y-axis) and their corresponding horizontal and vertical velocities (dotted lines, right y-axis). Data are shown for a control subject (A), an amblyopic subject without nystagmus (B), and an amblyopic subject with nystagmus (C). In the position traces, fast fixational eye movements (FEMs), such as micro-saccades (< 1°) in the control subject, fixational saccades (> 1°), and quick phases of nystagmus, appear as rapid step changes. In the velocity traces, these same events manifest as brief, high-amplitude velocity bursts. The inter-saccadic sections were defined as epochs of slow FEMs, i.e. inter-saccadic drifts in control and amblyopic subject without nystagmus and slow phases in amblyopic subject with nystagmus. Solid and dashed vertical lines represent the algorithmically detected saccade onsets and offsets, respectively, demonstrating that the Engbert algorithm successfully isolates discrete fast FEM events from slow inter-saccadic drifts and slow-phase nystagmus waveforms.
